# Splicing factor SRSF1 is essential for CD8 T cell function and host antigen-specific viral immunity

**DOI:** 10.3389/fimmu.2022.906355

**Published:** 2022-09-16

**Authors:** Ignacio Juarez, Shi Su, Zachary T. Herbert, John R. Teijaro, Vaishali R. Moulton

**Affiliations:** ^1^ Department of Medicine, Beth Israel Deaconess Medical Center, Harvard Medical School, Boston, MA, United States; ^2^ Department of Immunology, Ophthalmology and ENT, Faculty of Medicine, Complutense University of Madrid, Madrid, Spain; ^3^ Cardiovascular Institute, Beth Israel Deaconess Medical Center, Harvard Medical School, Boston, MA, United States; ^4^ Molecular Biology Core Facilities at Dana-Farber Cancer Institute, Boston, MA, United States; ^5^ Department of Immunology and Microbiology, The Scripps Research Institute, La Jolla, CA, United States

**Keywords:** immune response, T cells, cytokines, SRSF1, viral infection

## Abstract

Cytotoxic CD8 T cells are crucial for the host antigen-specific immune response to viral pathogens. Here we report the identification of an essential role for the serine/arginine-rich splicing factor (SRSF) 1 in CD8 T cell homeostasis and function. Specifically, SRSF1 is necessary for the maintenance of normal CD8 T lymphocyte numbers in the lymphoid compartment, and for the proliferative capacity and cytotoxic function of CD8 T cells. Furthermore, SRSF1 is required for antigen-specific IFN-γ cytokine responses in a viral infection challenge in mice. Transcriptomics analyses of Srsf1-deficient T cells reveal that SRSF1 controls proliferation, MAP kinase signaling and IFN signaling pathways. Mechanistically, SRSF1 controls the expression and activity of the Mnk2/p38-MAPK axis at the molecular level. Our findings reveal previously unrecognized roles for SRSF1 in the physiology and function of cytotoxic CD8 T lymphocytes and a potential molecular mechanism in viral immunopathogenesis.

## Introduction

Cytotoxic CD8 T cells are a key component of cell-mediated immunity, crucial for host protection against viral infection. The importance of cytotoxic T lymphocytes in viral infections has taken on new significance in relation to the current coronavirus disease (COVID)-19 pandemic caused by the severe acute respiratory syndrome coronavirus (SARS-COV)-2 virus, where deficiencies in the CD8 T response are associated with higher morbidity and mortality, with an increased disease recurrence, as occurs in elderly or immunocompromised patients (1, 2). Besides maintenance of adequate CD8 T lymphocyte population numbers in physiologic conditions, their proliferative capacity, direct target cell killing, and their ability to secrete cytokines, including the antiviral mediator IFN-γ, are crucial in the defense against viral infections (3). However, the molecules and mechanisms underlying CD8 T cell homeostasis and function and in the antigen-specific immune response to viral infections are not fully known.

We have recently uncovered immune-related roles for the post-transcriptional regulator, serine arginine rich splicing factor 1 (SRSF1) in CD4 T cells and its significance in immune-mediated inflammatory disease in mice and humans (4–6). SRSF1 controls the expression of genes involved in T cell receptor signaling (4, 5), and contributes to the regulation of cytokine production (7). Deficiency of SRSF1 in T cells leads to aberrant CD4 T cell function and exacerbation of autoimmune inflammatory disease in mice (6). Furthermore, SRSF1 is indispensable for Treg homeostasis and function (8). However, the role of SRSF1 in CD8 T cells and in the host cell mediated immune response to viral pathogens is not known.

Here we demonstrate that SRSF1 is necessary for CD8 T cell homeostasis, proliferation, cytotoxicity and cytokine function. SRSF1 is essential for the CD8 T cell-mediated antigen specific antiviral IFN-γ response and viral clearance. SRSF1 controls the transcriptomic landscape of T cells during viral infection through the regulation of proliferation, IFN signaling and MAPK signaling pathways. Mechanistically, SRSF1 contributes to the control of the Mnk2/p38 MAPK axis which is important for cellular homeostasis and cytokine function. Our study identifies previously unknown roles of SRSF1 in CD8 T lymphocytes and its functional significance in the host immune response to viral pathogens.

## Results

### SRSF1 is necessary for CD8 T cell homeostasis, proliferation and cytotoxic function

**Figure 1:**
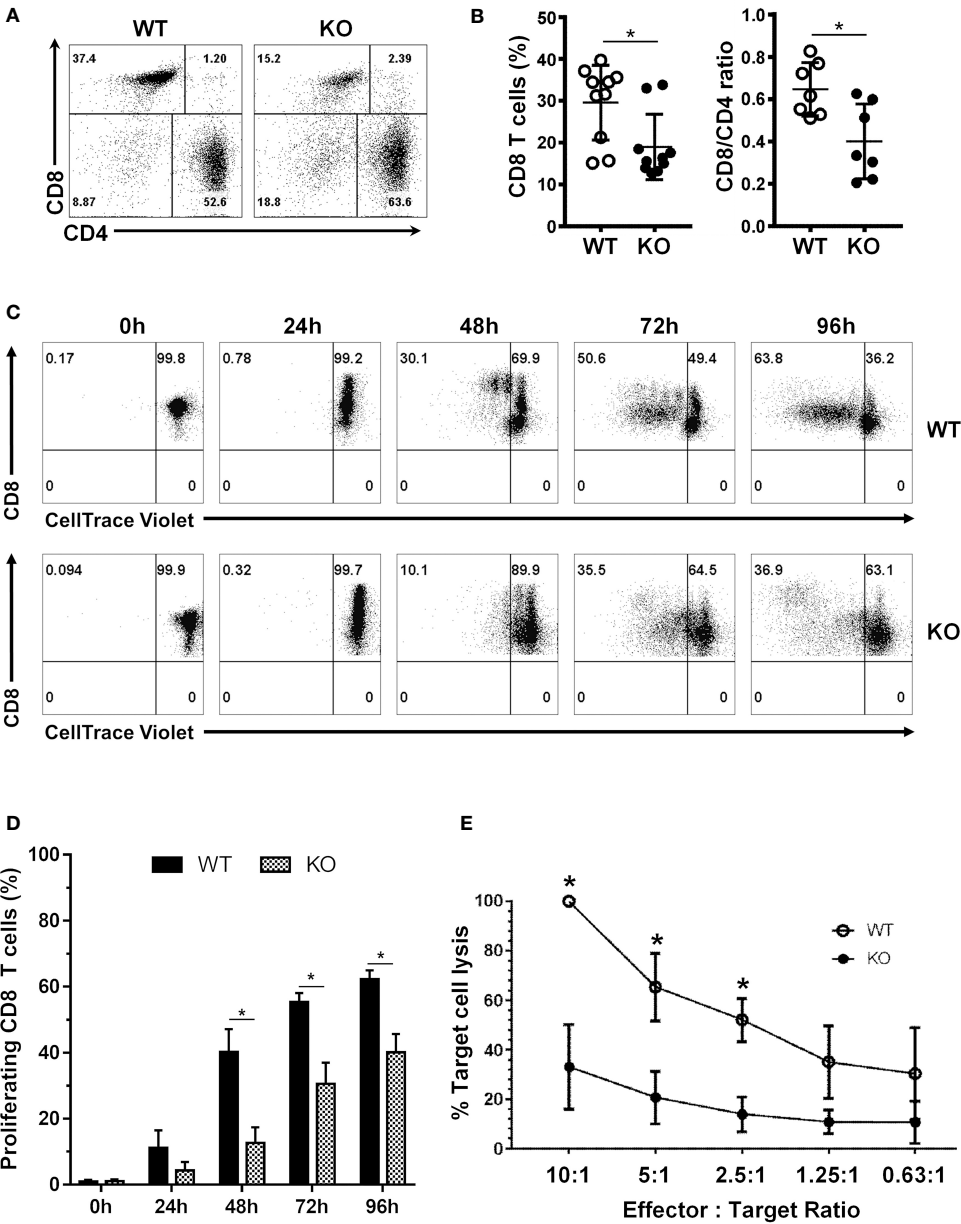
SRSF1 is necessary for CD8 T cell homeostasis, proliferation and cytotoxic function. **(A)** Flow cytometry dot plots show *ex vivo* frequencies of CD8 and CD4 T cells from the spleens of WT and *Srsf1*-cKO (KO) mice **(B)** Graphs show percentage of CD8 T cells and CD8/CD4 ratio (n=7). **(C)** Flow cytometry dot plots and histograms show proliferation of stimulated CD8 T cells from WT and *Srsf1*-cKO mice **(D)** Graphs show percentage of proliferated cells (n=5) **(E)** Graph shows percentage of target cell lysis after coculture with CD8 T cells from WT (n=3) or *Srsf1*-cKO mice (n=4), at different effector:target ratios. Graphs show mean ± SEM. p-values *<0.05.

We recently generated T cell conditional knockout mice (*Srsf1^flox/flox.dLck.Cre^*) that lack SRSF1 selectively in total T cells, by crossing Srsf1-flox/flox mice with distal Lck-Cre (dLck.Cre) mice. Since the distal Lck promoter (and hence the Cre recombinase) is expressed late during T cell development, predominantly in mature T cells, we previously confirmed that thymic development and generation of T cells in these mice is normal (6). Here, we examined the role of SRSF1 in CD8 T cells in these T cell-restricted Srsf1-conditional knockout (cKO) mice. While the frequencies of both CD4 and CD8 T cells were reduced in the peripheral lymphoid organs from Srsf1-cKO mice, the CD8 T cell compartment was profoundly depleted (18.9% n=10) with 2-3-fold reduced frequencies compared to the wild-type (WT) mice (29.6% n=10) (**Figures 1A, B**). The proportions of CD8:CD4 T cell populations were significantly altered from normal ratios of 0.64 in WT mice to 0.4 in the Srsf1-cKO mice (**Figure 1B**). We then examined the proliferative capacity of the CD8 T cells after T cell receptor (TCR) stimulation ex vivo. We found that the CD8 T cells from the Srsf1-cKO mice had a reduced proliferative capacity upon stimulation compared to those from WT mice (**Figures 1C, D**). We further evaluated the functional capacity of the CD8 T cells using the effector-to-target cell cytotoxicity luminescence assays. Srsf1-cKO CD8 T cells displayed significantly reduced ability to kill target cells as evidenced by decreased luminescent signals indicative of cell lysis in the 10:1, 5:1 and 2.5:1 effector:target ratios (p<0.05) compared to CD8 T cells from WT mice (**Figure 1E**). We also evaluated the cytotoxic capacity of the CD8 T cells by analyzing the presence of granzyme B and perforin cytotoxic granules in the CD8 T cells (**Supplementary Figure 5**). We found altered numbers of granzyme B (GzmB)+ and perforin+ CD8 T cells in the KO mice. Although the frequencies of GzmB+ CD8 T cells were higher and frequencies of Perforin+ CD8s not significantly different in spleens of KO mice, the absolute numbers of both Gzm B+ and Perforin+ CD8 T cells were significantly decreased in the KO mice (**Supplementary Figures 5B, D**). In the lymph nodes, no significant differences were observed although Perforin+ CD8 T cells tended towards a decrease in the KO mice (**Supplementary Figures 5B, D**). Altogether, these results indicate that SRSF1 is essential for the homeostasis, proliferative capacity, and cytotoxic function of CD8 T cells.

### SRSF1 is essential for the antigen specific cell mediated immune response to viral infection

**Figure 2:**
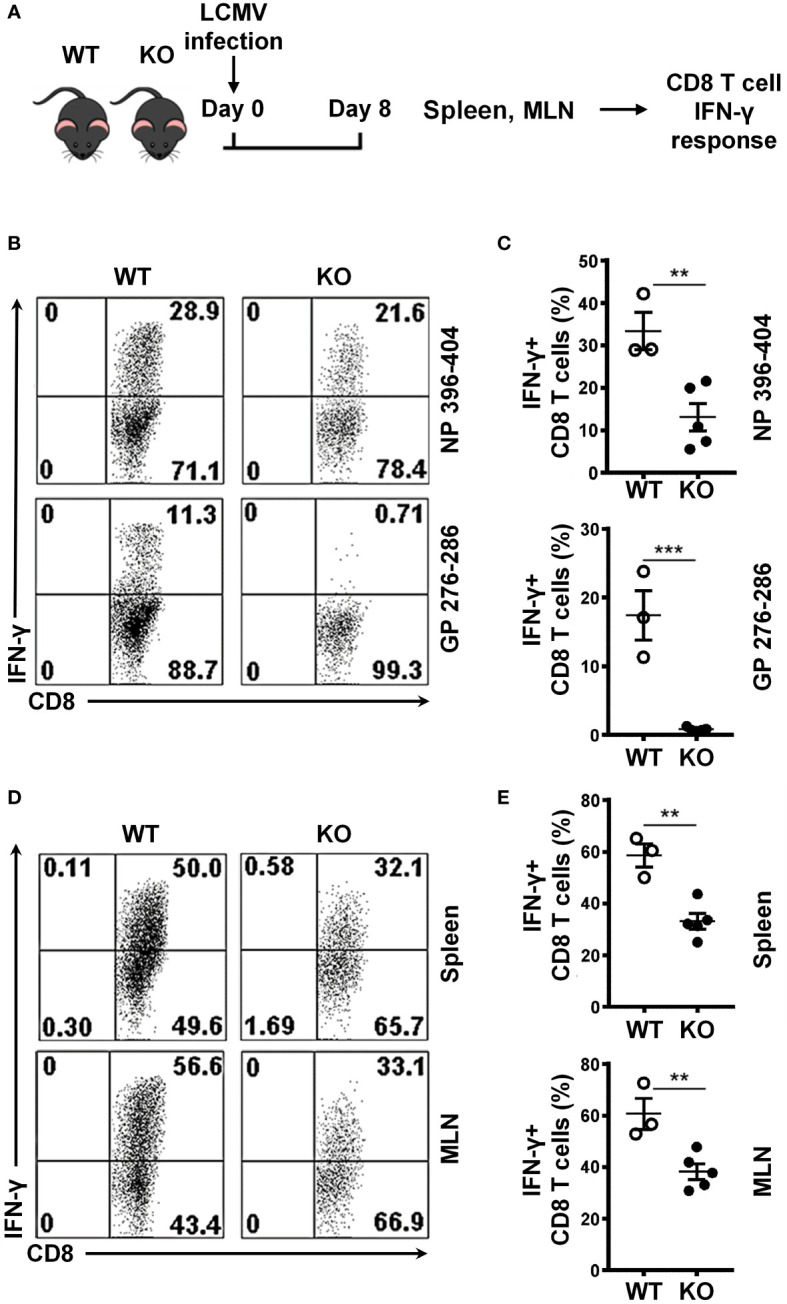
SRSF1 is essential for the antigen specific cell mediated immune response to viral infection. **(A)** Schematic presentation of LCMV infection protocol. *Srsf1*-cKO (KO) or WT mice were infected with LCMV Armstrong strain by IP injection. After 8 days of infection, spleen and mesenteric lymph nodes (MLN) were collected for isolation of lymphoid cells **(B)** Plots show IFN-γ+ CD8 T cells from spleen cells stimulated with LCMV peptides NP 396-404 or GP 276-286 gated on Thy1.2+ T cells. **(C)** Graphs show the percentages of IFN-γ+ CD8 T cells from WT (n=3) and KO (n=5) mice. **(D)** Plots show IFN-γ+ CD8 T cells from spleen and MLN cells stimulated with PMA and Ionomycin. **(E)** Graphs show the percentage of IFN-γ+ CD8 T cells from WT (n=3) and KO (n=5) mice. Plots show mean ± SEM. p-values **<0.01, ***<0.005.

To evaluate the role of SRSF1 in the cell-mediated immune response in viral infection, we infected WT and Srsf1-cKO mice with the lymphocytic choriomeningitis virus (LCMV)-Armstrong strain in an acute infection model (**Figure 2A**) using established protocols (9). On day 8 post infection, we evaluated spleen and mesenteric lymph node (MLN) cells for the host immune response to the infection. We examined the antigen specific IFN-γ responses of the CD8 T cells using two different LCMV-specific peptides, namely NP 396-404 and GP 276-286, in ex vivo stimulation assays. We found that Srsf1-cKO mice had significantly lower frequencies of IFN-γ+ CD8 T cells, (13.1% ± 3.3 for GP-276-286) and 0.8% ± 0.12 for NP-396-404), compared to the WT mice (33.4% ± 4.4 for GP-276-286 and 17.4 ± 3.6 for NP-396-404) (**Figures 2B, C**). Furthermore, stimulation of the spleen and MLN cells with PMA and Ionomycin, which bypasses the proximal T cell receptor signaling, still led to impaired production of IFN-γ in Srsf1-cKO CD8 T (33.1% ± 3.0 IFN-γ+ CD8 T cells in spleen, and 38.3% ± 3.0 in MLN) compared to WT mice (58.5% ± 4.5 and 60.6% ± 6.1, in spleen and MLN, respectively), suggesting that the defect in Srsf1-cKO T cells is within the downstream intracellular signaling components distal to the TCR (**Figures 2D, E**). Srsf1-cKO CD4 T cells did not showed any difference in the response to LCMV-peptides, although their activation in response to PMA-ION activation is lower than in WT mice (**Supplementary Figure 1**).

### Impaired expansion of Srsf1-cKO CD8 T cells during the immune response to viral infection

**Figure 3:**
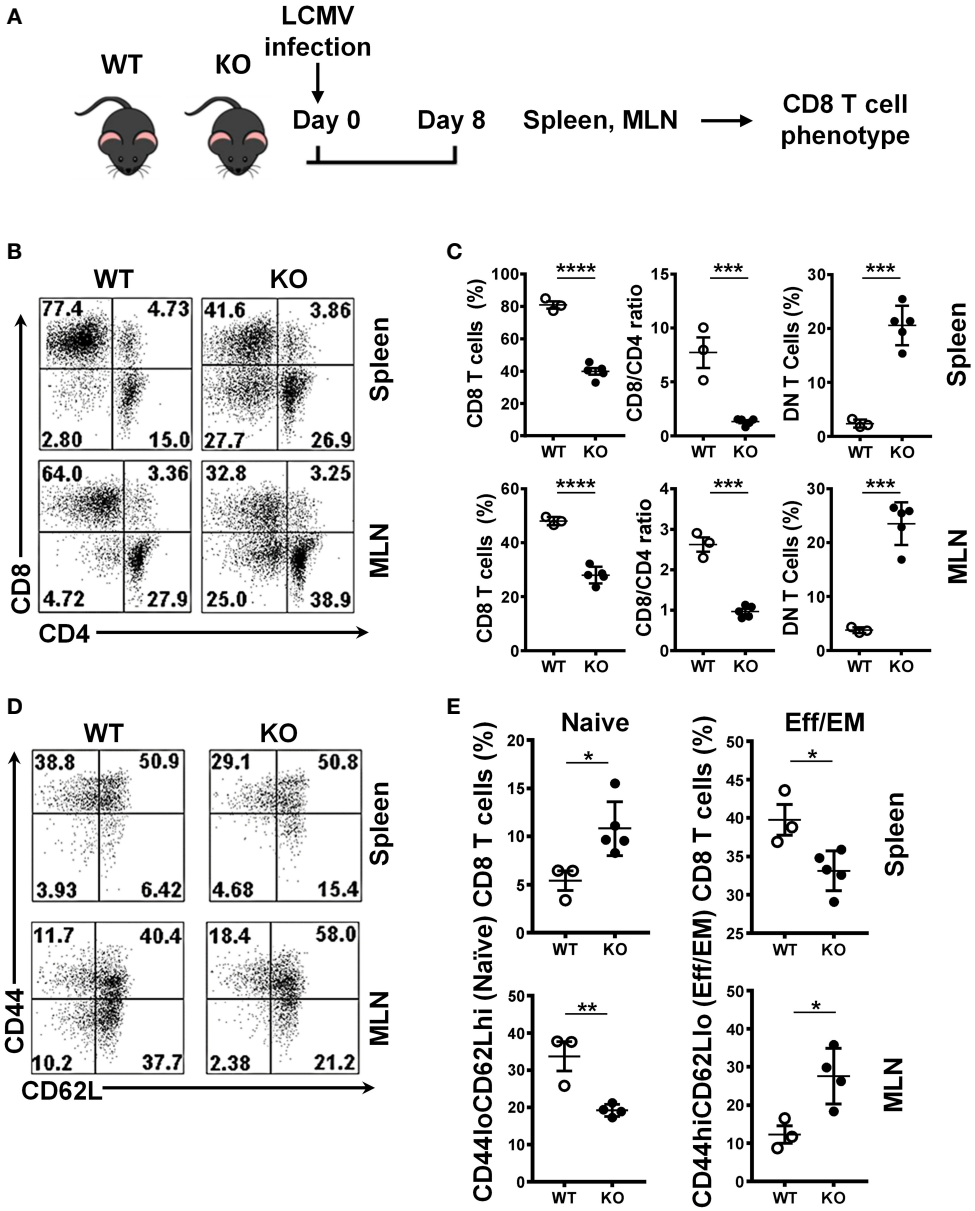
Impaired expansion of Srsf1-cKO CD8 T cells during the immune response to viral infection. **(A)** Schematic presentation of LCMV infection protocol. *Srsf1*-cKO (KO) or WT mice were infected with LCMV Armstrong strain by IP injection. After 8 days of infection, spleen and mesenteric lymph nodes (MLN) were collected for T cell phenotyping **(B)** Plots show the frequency of CD8, CD4 and CD4-CD8- double negative (DN) T cells gated on live Thy1.2+ T cells. **(C)** Graphs show the percentages of CD8 T cells, CD8/CD4 ratio and DN in the spleen and MLN of WT (n=3) and KO (n=5) mice. **(D)** Plots show the CD44 and CD62L staining of spleen and MLN cells gated on live CD8 T cells. **(E)** Graphs show the percentages CD44loCD62Lhi (naive) and CD44hiCD62Llo (effector) CD8 T cells from WT (n=3) and KO (n=5) mice. Graphs show mean ± SEM. p-values *<0.05 **<0.01, ***<0.005, ****<0.001.

To evaluate the role of SRSF1 in CD8 T cells in response to viral infection, we assessed the CD8 T cell populations in the LCMV-infected WT and Srsf1-cKO mice (**Figure 3A**). We found that Srsf1-cKO mice had a lower frequency of CD8 T cells in the spleen (33.2%) and MLN (28.0%, n=5) compared to the WT mice (48.1% and 51.3%, p=0.0001 and p<0.0001, respectively, n=3). The low number of CD8 cells was not accompanied by CD4 lymphopenia, as shown by the decreased CD8/CD4 ratio in the spleen (7.7) and MLN (2.6, n=3) of Srsf1-cKO mice compared to WT mice (1.3 and 0.96, p=0.0009 and p<0.0001, respectively) (**Figures 3B, C**). We also observed a significant increase of double negative (DN) T cells in Srsf1-cKO mice spleen (20.6%) and MLN (23.5%) compared to WT mice (2.4% and 3.8% respectively, p=0.0002 in both cases) (**Figures 3D, E**). The absolute numbers of CD8 and CD4 T cells corroborate the results of the frequency data (**Supplementary Figure 2**). These results show that deficiency of SRSF1 may lead to an impaired expansion of CD8 T cells during the viral immune response.

We also evaluated the differentiation state of CD8 T cells by CD44 and CD62L cytometry staining. We found a higher frequency of naive (CD44loCD62Lhi) CD8 T cells and a lower frequency of the effector (CD44hiCD62Llo) CD8 T cells in the spleen of the Srsf1-cKO mice compared to WT, and the reverse scenario when we performed the same staining in MLN cells, with a lower frequency of naïve and higher frequency of effector CD8 T cells, both after LCMV infection (**Figures 3D, E**), reflecting an impaired activation/generation of effector T cells in response to an acute viral infection.

### SRSF1 is necessary for CD8 T cell survival and viral clearance

**Figure 4:**
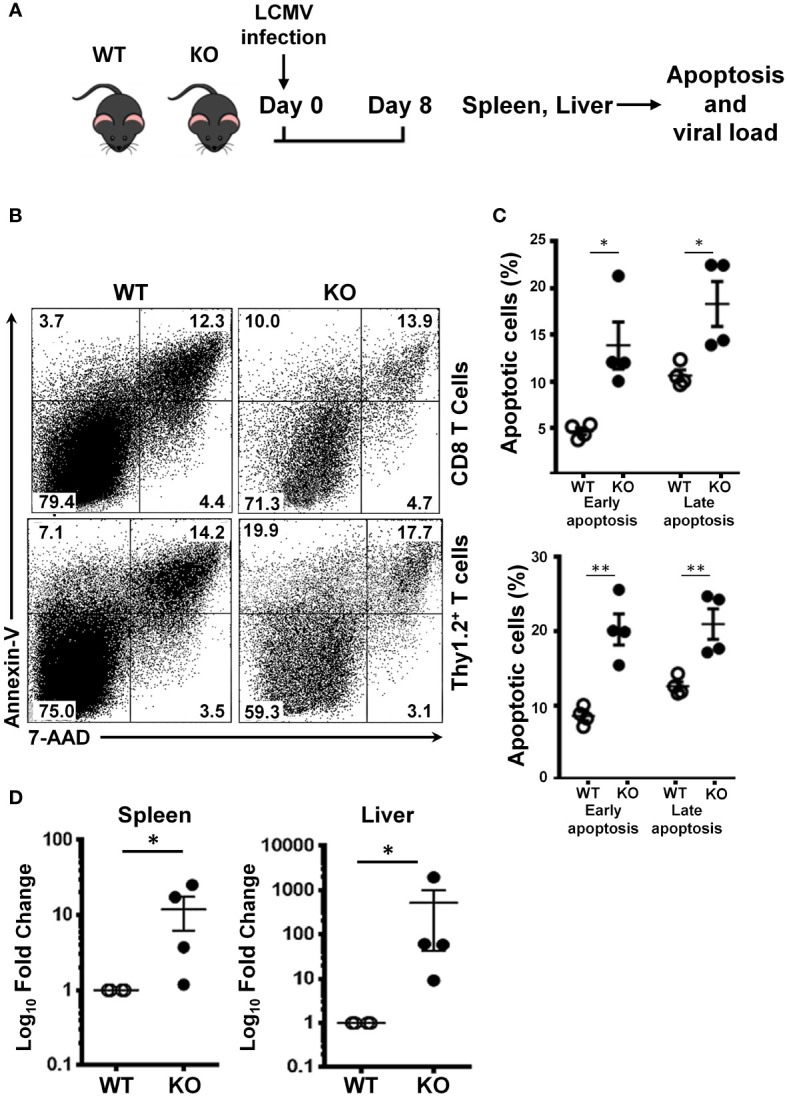
SRSF1 is necessary for CD8 T cell survival and viral clearance. **(A)** Schematic presentation of LCMV infection protocol. *Srsf1*-cKO (KO) or WT mice were infected with LCMV Armstrong strain by IP injection. On day 8 post infection, spleen and mesenteric lymph nodes (MLN) were collected for analysis of apoptotic cells. Spleen and liver were evaluated for LCMV viral load. **(B)** Plots show apoptotic cells gated on CD8 and Thy1.2+ T cells from spleens of mice infected with LCMV (n=4/group) **(C)** Graphs show frequencies of early (Annexin V+ 7-AAD-) and late Annexin V+ 7-AAD+) apoptotic cells. **(D)** LCMV GP mRNA expression in spleen and liver cells was measured by real-time RT-qPCR. Graphs show relative expression (n=4 mice/group). Graphs show mean ± SEM. p-values *<0.05 **<0.01.

Impaired proliferation and cell cycle arrest eventually lead to cellular apoptosis. It is known that SRSF1 is important for cell cycle progression, and its deficiency leads to impaired proliferation. Therefore, we examined whether SRSF1 controls the anti-apoptotic effects in T cells during the antiviral response. We measured the frequencies of early and late apoptotic T cells post LCMV infection (**Figure 4A**). We found that total T cells and CD8 T cells from the spleen of Srsf1-cKO mice had higher frequencies of early (Annexin V+ 7-AAD-) (20.3% and 13.9%, respectively) and late (Annexin V+ 7-AAD+) (21.1% and 18.3%, respectively) apoptotic cells compared to those from the WT mice (early 13.9% and 4.7%, late 18.3% and 10.7%, respectively, p<0.01 and p<0.05, respectively) (**Figures 4B, C**).

In addition to the CD8 T cell defects herein explained, we also quantified by qPCR the LCMV viral load in the spleen and liver, two of the main target tissues of this virus. We found that the Srsf1-cKO mice had significantly higher LCMV viral loads (10-fold in spleen and 100-fold in liver) compared to WT mice after 8 days of LCMV infection (**Figure 4D**). This data suggests an impaired viral clearance ability of the Srsf1-cKO mice.

### Transcriptomic profiling reveals that SRSF1 controls antiviral cytokine signaling and apoptotic pathways in T cells

**Figure 5:**
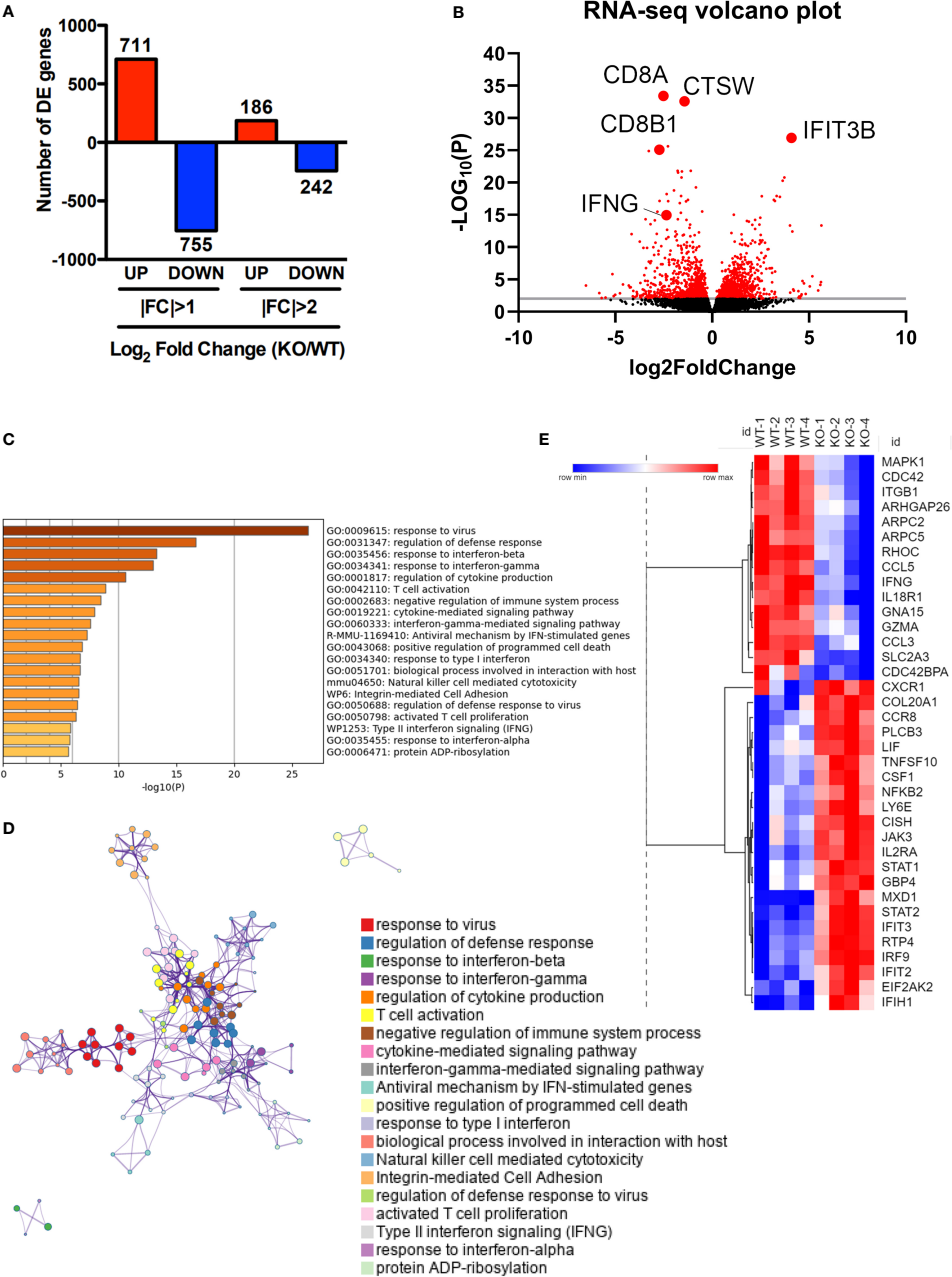
Transcriptomic profiling reveals that SRSF1 controls antiviral cytokine signaling and apoptotic pathways in T cells. Total T cells were isolated from spleens of LCMV infected WT and KO mice (n=4/group). **(A)** RNA-sequencing data analysis shows differentially expressed **(D, E)** genes with log_2_fold change **(F, C)** differences at p<0.05. **(B)** Volcano plot showing upregulated and downregulated genes in KO T cells after LCMV infection compared to the WT. **(C)** Bar graph of top 40 enriched terms, colored by p-values. **(D)** GO terms enrichment map shows clusters of top 40 pathways. **(E)** Heat map showing average expression of selected DE genes associated with enriched terms.

To better understand how SRSF1 affects the biological processes in the context of viral infection, we performed RNA sequencing of spleen T cells from WT and Srsf1-cKO mice on day 8 post LCMV infection. Our transcriptomics data show that Srsf1 controls a large number of genes during the antiviral immune response. A total of 1894 genes were differentially expressed (DE) in KO compared to WT T cells, with 711 genes upregulated and 755 genes downregulated at the 2-fold cutoff and 186 genes up and 242 genes down at the 4-fold cutoff (p < 0.05) (**Figure 5A**). The volcano plot shows the DE genes in the T cells from Srsf1-cKO mice compared to WT mice (**Figure 5B**). Gene Ontology (GO) terms and Kyoto encyclopedia of genes and genomes (KEGG) enrichment analysis revealed pathways involved in response to IFN-γ and its mediated signaling pathway, T cell activation, cytokine-mediated signaling, and regulation of cytokine production (**Figures 5C, D**). Several genes associated with the enriched terms in the pathway analysis were downregulated in Srsf1-cKO mice, including those in the MAPK pathway (**Figure 5E**). These findings suggest that SRSF1 controls genes and molecular pathways important for the host antiviral immune response. RNA-seq expression data and GO and KEGG analysis have been included as supplementary material (**Supplementary Tables 1** and **2**, respectively).

### SRSF1 controls Mnk2 expression and activity of the p38 MAPK pathway

**Figure 6:**
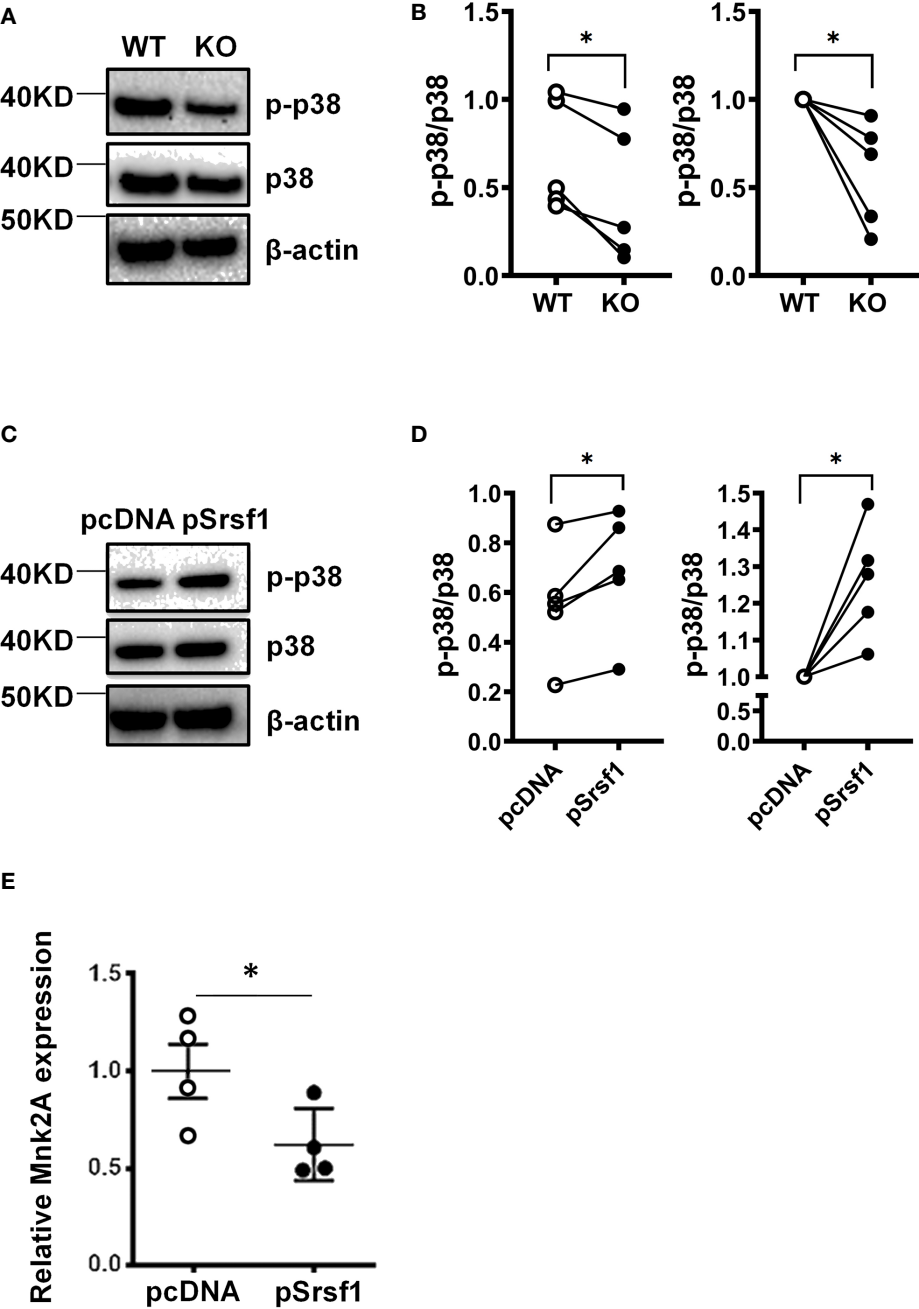
SRSF1 controls Mnk2 expression and activity of the p38 MAPK pathway. **(A)** Total mouse T cells were isolated from spleens of WT and *Srsf1*-cKO mice and stimulated with anti-CD3 and anti-CD28. Total protein was immunoblotted for phospho-p38 (p-p38), p38, and β-actin. Image showing one representative blot from 5 independent experiments. **(B)** Graph shows relative quantitation by densitometry. **(C)** Peripheral blood T cells were isolated from healthy donors and transfected with empty vector (pCDNA) or Srsf1 overexpression plasmid (pSrsf1). Transfected T cells were stimulated with anti-CD3 and anti-CD28. Total protein was immunoblotted for phospho-p38 (p-p38), p38, and β-actin. Image showing one representative blot from 5 independent experiments. **(D)** Graph shows relative quantitation by densitometry. **(E)** Relative mRNA expression of Mnk2a isoform in human T cells. Graphs show mean ± SEM, p-values *<0.05.

Based on its roles in cellular proliferation/apoptosis and cytokine signaling, we investigated the relationship between SRSF1 and activity of the p38-MAPK signaling pathway. We stimulated spleen T cells with CD3/CD28 and examined the expression of phosphorylated p38 protein expression levels. We found that activated Srsf1-cKO T cells had lower levels of phosphorylated p38 protein expression compared to WT T cells (**Figures 6A, B**). To evaluate if SRSF1 is sufficient to modulate the p38 pathway, and to test this in human T cells, we overexpressed SRSF1 by transient transfection of Srsf1-expression plasmid vector in peripheral blood T cells, using optimized conditions for SRSF1 overexpression (**Supplementary Figure 4**), and assessed the expression of phosphorylated p38 protein. We found an increase of phosphorylated p38 expression in T cells overexpressing SRSF1, compared to control transfected cells (**Figures 6C, D**). As recent studies have suggested that alternatively spliced isoforms of the Mnk2 kinase can modulate the p38-MAPK pathway (10), we hypothesized that SRSF1 controls the expression of Mnk2, which further controls the activation of p38. We overexpressed SRSF1 in T cells and measured the mRNA expression of Mnk2a, the isoform of Mnk2 that activates p38 mediated cell death. We found that cells overexpressing Srsf1 had significantly lower Mnk2a mRNA levels (**Figure 6E**). Our results suggest that SRSF1 controls the Mnk2/p38-MAPK axis which may contribute to its role in cell proliferation/survival and cytokine function.

## Discussion

In this study, we reveal that the absence of Srsf1 caused a marked decrease in the CD8 T cell population in mice, as evidenced by the low frequency of these cells in the spleen and MLN. The low numbers of CD8 T cells were a result of both defective proliferation and increased apoptosis. The CD8 T cells from Srsf1-cKO mice also have defective cytotoxic capacity. When the Srsf1-cKO mice were challenged with acute LCMV infection, their CD8 T cells exhibited reduced IFN-γ production capacity in response to re-stimulation with LCMV-specific peptides as well as decreased differentiation of naïve CD8 T cells into effector memory T cells. Our transcriptomics data provides information of the altered gene expressions and their associated pathways including cytokine production, T cell activation and IFN signaling in Srsf1-cKO mice post LCMV infection. Mechanistically, SRSF1 controls the expressions and activity of several signaling pathways, such as the Mnk2-p38-MAPK signaling axis. Our study shows that SRSF1 plays a crucial role in CD8 homeostasis and function and in the regulation of host antiviral immunity.

In this study, we identified previously unrecognized roles for Srsf1 as one of the main molecules involved in the development of an effective CD8 T cell response, since its absence leads to defects in the homeostasis and differentiation of CD8 T lymphocytes, both in basal conditions and in the context of a viral infection. This molecule has already been identified as a key post-transcriptional regulator in the late stages of thymocyte development (11). In our study, CD8 T cells lacking SRSF1 are unable to generate an effective response to LCMV, limiting their maturation and differentiation into memory cells, thus failing to respond to LCMV-specific peptides. The LCMV infection model is compatible with other RNA +SS viruses, such as HIV and HCV (12), so our results could theoretically be extrapolated to these types of infections, although other types of infections, such as bacterial infections, would require more specific studies focused on these pathogens, since this type of response involves combined responses and depends on the type of pathogen involved in the infection.

The impairment of CD8 response may be mediated by a direct effect by the lack of SRSF1 in the CD8 T cells, but also could be exacerbated as all T cells lacks SRSF1, impairing the coordinated anti-viral responses of these mice. The findings of this study could shed light to the potential cause as to why patients with immune-mediated diseases who have low Srsf1 expression in their T lymphocytes maybe more susceptible to viral infections. The deleterious effect of the lack of SRSF1 on CD8 T cells in these mice implies a defect in the proliferation of these cells, which could be related to other studies implicating SRSF1 in cell cycle progression in different types of tumors (13, 14). Although in this work we see a lack of proliferation of CD8 T cells, this could be due to the role of SRSF1 in cell cycle progression, which would have the ultimate effect of decreasing proliferation. This is the first time that SRSF1 is studied in the context of T cell responses against viral infection, including the role of SRSF1 in cell cycle progression and proliferation of T cells in an inflammatory microenvironment caused by a viral infection. These results provide the basis for future work to determine which pathways are most relevant in this context.

After a viral infection, such as LCMV, the kinetics of infection results in a viral load change that peak on day 5 and diminishes on day 8, corresponding to the course from primo-infection to complete resolution (15). This viral kinetics is accompanied by an increase in the population of cytotoxic T cells (CD8 T cells), peaking on day 8 post infection (16). The Srsf1-cKO mice failed to maintain this kinetics after LCMV infection, with high viral loads in their spleen and liver on day 8, accompanied by an inefficient CD8 T cell response that they not only failed to expand but more importantly, exhibited a complete absence of CD8 T cells responding to viral antigens. It is also interesting how SRSF1-cKO CD8 T lymphocytes also showed increased apoptosis during antiviral response, a result that is in agreement with previous data from our group that show that the absence of SRSF1 in mature T cells increases apoptosis of T cells *in vitro*, and furthermore, overexpression of SRSF1 rescues this and increases survival of T cells (17). Interestingly, we found that although the KO CD8 T cells exhibited the presence of GzmB and Perforin, there was a significant decrease in the absolute numbers of GzmB+ and Perforin+ CD8 T cells in the KO mice. As the immune response depends on the population size of the CD8 cytotoxic T cells, these reductions can contribute to the compromised cell-mediated response to the viral infection. Although the production of these cytotoxic granules was not altered, SRSF1 has been linked to genes involved in impaired cytotoxic granule exocytosis by lymphoblasts and NK cells (18, 19). Therefore, it may be possible that the absence of SRSF1 in CD8 T lymphocytes may also affect granule exocytosis, limiting the cytotoxic function of CD8 T cells, however these would require further studies to evaluate these possibilities.

Therefore, these results confirm this data and bring it into the context of a viral infection. RNAseq data also shows an important alteration of pathways that mediate the response against viral infection, such as pathways related to IFN (type I and II) response, T cell activation and proliferation and cytokine production, which, consistent with functional data, places SRSF1 as an important mediator of the response to viral infections. Interestingly, we found no differences in gene expression in resting CD8 T lymphocytes in unstimulated conditions (data not shown), as reported in our previous study (6). Therefore, the differences shown here likely depend on the role of SRSF1 during activation of T lymphocytes after viral infection.

In this work, we also explore a mechanistic pathway for the involvement of SRSF1 in viral infections. The kinase Mnk2 controls the phosphorylation of p38, one of the main proteins involved in the MAPK pathway (10). In humans, the Mnk2a splicing isoform has the MAPK binding domain and activates p38 (20). Non-canonical p38 activation occurs in T-lymphocytes upon antigen presentation (21). The pro-survival mechanisms of p38 involve the modulation of alternative splicing through MNK1/2 (22). Here we have shown that SRSF1 controls the expression of Mnk2a isoform. To the best of our knowledge, this is the first time that the Srsf1-MNK2-p38 pathway was linked to T cell response, more specifically, to IFN-γ production. Other works have described that the pharmacological blockade of p38 leads to an exacerbated proliferation in CD8 T cells (23). Considering the role of p38 in T cell function (24–26), the compromised p38 signaling pathway in SRSF1-KO T cells may be a crucial contributor to the T cell defect in the KO mice and impact their ability to clear the virus. Therefore, increasing p38 activation in the KO T cells either ex vivo or by *in vivo* strategies may be successful in reversing the phenotype of CD8+ T cells in the KO mice. However, p38 expression data indicate that there is an increased expression of this kinase in activated T cells from SRSF1-KO mice (data not shown), having a lower phosphorylation dependent on this activation as shown in **Figure 6**. This increase in p38 expression in the absence of SRSF1 is concordant with computational data obtained using SEEK software (https://seek.princeton.edu/seek/), which show that there is a small inverse coexpression relationship between MAPK11 (p38-β) and SRSF1. Therefore, we think that future studies focusing on the relationship of SRSF1 and p38 in relation to the CD8 T cell immune response could be of interest to determine the functioning of this pathway.

Interestingly, we observed higher frequencies of naive (CD44loCD62Lhi) CD8 T cells and a lower frequency of the effector (CD44hiCD62Llo) CD8 T cells in the spleen of the Srsf1-cKO mice post infection. However, in the MLNs of KO mice we observed an increase in effector/effector memory CD8 T cells in the KO mice compared to WT mice. This may in part be explained by the defects observed in the activation and proliferation of CD8 T cells in the KO mice in response to polyclonal T cell stimuli (anti-CD3/CD28 and PMA/ION) or with LCMV antigen-specific peptides. Normally, CD8 T cells in WT mice expand to reach a peak burst size at day 8, after which they enter a decay phase after complete clearance of the virus (27), whereas in the KO mice the defects in activation and proliferation of cytotoxic cells may delay viral clearance, which might explain a higher proportion of residual effector cells in the lymph nodes, in contrast to WT mice. Additionally, it has also been reported that MLN T cells could present abnormal phenotypes under viral infection (28). Is also important to remark that Srsf1-cKO mice have a disrupted naïve to effector/effector memory phenotype, as we already have demonstrated (8), which can be independent of LCMV infection. Nevertheless, our data suggest that there was a defective naive and effector CD8 T cell migration in the Srsf1-cKO mice post infection. Interestingly, we found a population of double negative (DN) T cells in the spleen of mice, both at baseline (**Figure 1**) and after LCMV infection. These DN T cells still present several unknowns regarding their function and development. However, some studies have shown that these cells are derived from CD8+ T cells (29, 30). This population could be of great interest for studies focused on the function of these cells in viral infection.

A limitation of this study is that the Srsf1-cKO mice are a total T cell-specific knockout, which involves all the T cell subsets (Th1, Th2, Treg, etc.) that may affect CD8 T cell function and differentiation. A E8i-cre Srsf1-flox (mature CD8 T cell conditional Srsf1-cKO mice) is required to assess more precisely the role of SRSF1 in this population. In addition, our transcriptomics data provided an array of pathways that were altered in Srsf1-cKO mice in the context of LCMV infection. There may be other pathways that act in concert with the MAPK pathway, such as IFNα response and IL2/STAT5 signaling pathways. Future studies on these pathways would provide us more insights on the role of SRSF1 in host immunity. As the mechanisms involving SRSF1-MNK2-P38 pathway in the T-cell response is still unknown, we consider this as a good approach to study in future studies, and in the context of other viral or bacterial infections. SRSF1 is known to control the alternative splicing of a number of genes, which interconnect several signaling pathways, some of which are related to the T response in viral infections. Also, while this study focuses on the role of SRSF1 on the T cell-driven immune response to acute viral infection (modeled with the Armstrong strain of LCMV), and we have demonstrated significant differences in viral genetic material in the spleen and liver at 8 days of infection, it would be interesting to evaluate copies of the LCMV at earlier timepoints. Furthermore, it would be of interest to evaluate this same response in a chronic infection model, using the Clone-13 strain of LCMV. In the specific case of the MNK2/p38 pathway, SRSF1 regulates alternative splicing of MNK2 towards the MNK2a or MNK2b variant, leading to changes in the catalytic activity of the molecule and phosphorylation of p38 (10). SRSF1 has also been associated with proliferative pathways in different types of cancer (13), which would condition the expansion of CD8 T lymphocytes in response to viral infection, pathways in which the p38 is also involved. This is the first time that the effect of SRSF1 on CD8 T cells in the context of viral infection has been described, and our findings provide the basis for future work to determine which pathways are most relevant in this context, including those to define direct versus indirect binding targets of SRSF1 by RIP-seq or CLIP-seq types of RNA-protein binding studies to delineate which genes are direct targets and which are downstream and therefore indirect targets.

We have also seen that the response to the non-antigen-specific PMA/Ionomycin stimulus is lower in SRSF1-KO than in WT mice, and the dramatic lack of interferon gamma-producing cells, together with the reduced proliferation of CD8 T cells in KO mice, suggest that there is an intrinsic defect in CD8 T cell activation in SRSF1 KO mice, which in turn leads to a defect in the generation of antigen-specific CD8 T cells specific against LCMV. While we have demonstrated with the use of LCMV-specific peptides the antigen-specific responses in viral infection, future work would benefit from the use of CD8 tetramers or adoptive transfer of TCR transgenic T cells to further evaluate the effect of LCMV infection on antigen specific T cells *in vivo*.

In conclusion, we have demonstrated an essential role of splicing factor SRSF1 in CD8 T cell function and in the host antigen-specific immune response. These findings are of relevance for a better understanding of host immunity against viral infections.

## Materials and methods

### Mice

B6.Srsf1.flox/flox mice were generated (8) and B6.dLck.Cre (stock 012837) mice were purchased from the Jackson Laboratory (Bar Harbor, ME, USA). The two strains were crossed to generate the B6.dLck.Cre.Srsf1.flox/flox (Srsf1-cKO) strain. B6.dLck.Srsf1.flox/flox lacking dLCK-cre. Mice were maintained in a specific pathogen-free animal facility at Beth Israel Deaconess Medical Center (BIDMC). All studies were approved by the Institutional Animal Care and Use Committee.

### Antibodies and reagents

Flow cytometry antibodies and other reagents: anti-mouse CD4 (GK1.5), CD8a (53-6.7), CD90.2 (53-2.1), IFN-γ (XMG1.2), CD44 (IM7), CD62L (MEL-14), CD107a (1D4B), 7-AAD and Annexin-V (BioLegend, San Diego, CA, USA).

Western blot antibodies: anti-phospho-p38 MAPK (Thr180/Tyr182) (Cell Signaling) (D3F9), anti-beta-actin (AC-74) (Merck) and goat anti-rabbit IgG-horse-radish peroxidase (HRP) and goat anti-mouse IgG-HRP were from Thermo Fisher Scientific (Waltham, MA, USA) and Ammonium-Chloride-Potassium (ACK) lysing buffer was from Fisher Scientific (Pittsburgh, PA, USA).

### LCMV infection

The LCMV-Armstrong strain was a kind gift from Dr. John Teijaro (The Scripps Research Institute). Mice were infected *via* intraperitoneal injection (i.p.) with 10^5^ PFU and maintained for 8 days in the Animal Research facility at BIDMC. On day 8 post infection, mice were euthanized, and tissues were collected for downstream procedures (31).

### Tissue processing and cell isolation

Spleens and mesenteric lymph nodes were homogenized using a syringe plunger and 70μm cell strainer. RBC lysis was performed with ACK lysing buffer for 3 min. All cell cultures were in RPMI complete medium (RPMI plus 10% FBS plus penicillin and streptomycin antibiotics).

### Flow cytometry

Zombie aqua viability dye was used for live/dead cell staining. Surface staining was performed in FACS staining buffer (PBS plus 2% FBS) on ice for 30 minutes with Fc block. For IFN-γ production, cells were stimulated for 4 hours in culture medium with PMA (100 ng/mL), ionomycin (1 μM), and monensin (1 μL/mL) or with the specific LCMV peptides NP396–404 and GP 276–286 (RP20090 and RP19983, GenScript, Piscataway, NJ, USA). Cells were surface stained, followed by fixation and permeabilization using the BD intracellular staining kit (BD Biosciences) for intracellular staining. Flow cytometry data were acquired on a BD LSRII or CytoFLEX LX and analyzed with FlowJo software. All procedures were performed according to the manufacturer’s instructions.

### Cytotoxicity assays

CD8+ T cells were isolated from Srsf1fl/fl and Srsf1fl/fl.dLckcre mice using MACS as CD8+ effector T cells. The murine lymphoblastoid T cell line EL4 was cultured in DMEM complete medium (supplemented with 10% FBS and 1% penicillin/streptomycin) and cocultured with the isolated CD8+ effector T cells with the following Effector : Target ratios: 10:1, 5:1, 2.5:1, 1.25:1 and 0.63:1. The cells were incubated at 37°C with 5% CO2 for 4 hours to induce cell cytotoxicity. The cytotoxicity was measured using the CytoTox 96 Non-radioactive cytotoxicity assay LDH detection kit (Promega) according to the manufacturer’s instructions.

### Proliferation assays

Spleen cells from mice were labeled using the CellTrace™ Violet Cell Proliferation Kit (Thermo Scientific) following manufacturer’s instructions. 3 million cells were plated in a 24 well plate and stimulated with anti-CD3 (2 μg/mL) and anti-CD28 (2 μg/mL). Cells were collected at 24, 48, 72 and 96 hours after stimulation and analyzed by flow cytometry.

### RNA-sequencing

Total T cells were isolated from spleens by magnetic assisted cell sorting (MACS), using the Pan T cell isolation kit (Miltenyi Biotech). Total RNA was extracted using the RNeasy mini kit (Qiagen) and submitted for RNA sequencing to the Molecular Biology Core Facilities at the Dana-Farber Cancer Institute (DFCI). Libraries were prepared using Roche Kapa mRNA HyperPrep strand specific sample preparation kits from 200ng of purified total RNA according to the manufacturer’s protocol on a Beckman Coulter Biomek i7. The finished dsDNA libraries were quantified by Qubit fluorometer and Agilent TapeStation 4200. Uniquely dual indexed libraries were pooled in an equimolar ratio and shallowly sequenced on an Illumina MiSeq to further evaluate library quality and pool balance. The final pool was sequenced on an Illumina NovaSeq 6000 targeting 40 million 150bp read pairs per library at the Dana-Farber Cancer Institute Molecular Biology Core Facilities. Sequenced reads were aligned to the UCSC mm10 reference genome assembly and gene counts were quantified using STAR (v2.7.3a) (32). Differential gene expression testing was performed by DESeq2 (v1.22.1) (33). RNAseq analysis was performed using the VIPER snakemake pipeline (34).

### Transfections

Healthy donor deidentified blood samples were obtained from the Kraft donor center at Dana Farber Cancer Institute. All studies were approved by the institutional review board. T cells were isolated from blood using the Rosette Sep T cell isolation kit (Stem Cell Technologies). Human T cells were transfected using the Amaxa human T cell nucleofector kit (Lonza, Cologne, Germany), following the manufacturer’s instructions. Briefly, 3 × 10^6^ to 6 × 10^6^ cells were resuspended in 100 μL nucleofector solution. Plasmid DNA pcDNA3.1 empty vector or pcDNA3.1-Srsf1 (0.5 μg/10^6^ cells) was added, and cells were transferred into a cuvette and electroporated using the U-014 program in the nucleofector device. Cells were immediately rescued into prewarmed medium and cultured overnight.

### RT-qPCR

mRNA was isolated using the RNeasy mini kit (Qiagen) and reverse transcribed into cDNA using the ecodry oligo dT RNA to cDNA premix (Clontech). Real-time quantitative PCR amplification was carried out with SYBR Green I mastermix using a LightCycler 480 (Roche) instrument, following the program: initial denaturation at 95 °C for 5 min; 40 cycles of amplification (denaturation at 95 °C for 15 s, annealing at 60 °C for 15 s, extension at 72 °C for 30 s); one cycle of melting curves (95 °C for 15 s, 65°C for 2 min and 97 °C continuous), with a final cooling step at 37 °C. Threshold cycle values were used to calculate relative mRNA expression by the ΔCt relative quantification method. Primers were purchased from Eurofins Genomics. Primer sequences are:

Mnk2 forward 5’-GCTGCGACCTGTGGAGCCTGGG-3’.
Mnk2a reverse 5’-GATGGGAGGGTCAGGCGTGGTC-3’.
Mnk2b reverse 5’-GAGGAGGAAGTGACTGTCCCAC-3’.
LCMV-GP reverse 5’-GCAACTGCTGTGTTCCCGAAAC-3’.
LCMV-GP forward 5’-CATTCACCTGGACTTTGTCAGACTC-3’

### Western blot

Total protein was extracted using RIPA buffer (Boston Bioproducts) and electrophoresed on NuPAGE 4–12% Bis-Tris gels (Life Technologies). Proteins were transferred to PVDF membranes, blocked with 5% (wt/vol) non-fat milk in Tris-buffered saline with 0.05% Tween 20 (TBS-T) for 1 h, followed by an incubation with primary antibody 1:1000 dilution (1:10000 for β-actin antibody) in TBS-T 5% milk at 4 °C overnight (room temperature for 1h for β-actin antibody). Membranes were washed with TBS-T, incubated with HRP-conjugated secondary antibody for 1 h, washed with TBS-T, developed with enhanced chemiluminescence (ECL) reagents (1:4000 ECL prime; GE Healthcare). Retrieved bands were visualized by a ChemiDoc XRS imager (Bio-Rad). Densitometry was performed using Image Lab (Bio-Rad).

### Statistical analysis

Quantitative data were reported as the mean and standard error of the mean (SEM). The Student’s t test was used for continuous variables, and paired t test was used in the transfection experiments to determine the global tendency of individual experiments from different days. All calculations were performed using GraphPad Prism version 8.0 (GraphPad). p < 0.05 (2-sided) was considered statistically significant.

## Data availability statement

The data presented in the study are deposited in the Gene Expression Omnibus (GEO) repository, accession number GSE201028.

## Ethics statement

The studies involving human participants were reviewed and approved by Kraft donor center at Dana Farber Cancer Institute. The patients/participants provided their written informed consent to participate in this study. The animal study was reviewed and approved by Institutional Animal Care and Use Committee at BIDMC Animal Facility.

## Author contributions

IJ, SS, and VM designed the research; IJ, SS, and ZH performed research; JT and ZH contributed reagents/analytic tools; IJ, SS, ZH, and VM analyzed data; and IJ, SS, ZH, JT, and VM wrote the paper. All authors contributed to the article and approved the submitted version.

## Funding

This work was supported by a grant from the National Institutes of Health NIAMS (R01 AR068974) to VM and a Complutense University, Margarita Salas grant (young PhDs, CT31/21) to IJ.

## Acknowledgments

Authors acknowledge Michael Cassidy for help with data analysis.

## Conflict of interest

The authors declare that the research was conducted in the absence of any commercial or financial relationships that could be construed as a potential conflict of interest.

## Publisher’s note

All claims expressed in this article are solely those of the authors and do not necessarily represent those of their affiliated organizations, or those of the publisher, the editors and the reviewers. Any product that may be evaluated in this article, or claim that may be made by its manufacturer, is not guaranteed or endorsed by the publisher.

## Supplementary material

Supplementary Figure 1 SRSF1-KO CD4 T cells respond normally to LCMV peptides. **(A)** Plots show IFN-γ+ CD4 T cells from spleen cells stimulated with LCMV peptides NP 396-404 or GP 276-286 gated on Thy1.2+ T cells.

Supplementary Figure 2 CD8 and CD4 T cells numbers after LCMV infection (day 8). Graphs show the total numbers of CD8 and CD4 T cells in the spleen WT (n=3) and KO (n=5) mice. CD8 total number are depleted in KO mice (0.8 ± 0.2) compared to WT mice (16.6 ± 4.0) Graphs show mean ± SEM. p-values **: <0.01.

Supplementary Figure 3 Unstimulated control WB of Figure 6. **(A)** Total T cells were isolated from spleens of WT and Srsf1-cKO mice and, without stimulation, total protein was immunoblotted for phospho-p38 (p-p38), p38, and β-actin. Image showing two representative blot from 5 independent experiments. **(B)** Peripheral blood T cells were isolated from healthy donors and transfected with empty vector (pCDNA) or Srsf1 overexpression plasmid (pSrsf1). Unstimulated transfected T cells total protein was immunoblotted for phospho-p38 (p-p38), p38, and β-actin. Image showing two representative blot from 5 independent experiments.

Supplementary Figure 4 SRSF1 overexpression in human T cells. Peripheral blood T cells were isolated from healthy donors (HD) and transfected with empty vector (pcDNA) or Srsf1 overexpression plasmid (pSrsf1). Total protein was immunoblotted for SRSF1 and β-actin.

Supplementary Figure 5 Granzyme B+ and Perforin+ CD8 T cells are altered in SRSF1-KO mice following acute LCMV infection. **(A)** Flow cytometry dot plots show intracellular staining of granzyme B (GzmB) in CD8 T cells from the spleen and MLN of WT and SRSF1-KO (KO) mice. **(B)** Graphs show average frequencies of GzmB+ CD8 T cells in the spleen and MLN, as well as absolute numbers of GzmB+ CD8 T in spleens of WT and KO mice. **(C)** Flow cytometry dot plots show intracellular perforin staining in CD8 T cells from spleen and MLN of WT and KO mice. **(B)** Graphs show average frequencies of perforin+ CD8 T cells in the spleen and MLN, as well as absolute numbers of GzmB+ CD8 T cells in spleen, of WT and KO mice. ** = p<0.01; *** = p<0.001.
