# Supplementary figures and images for: Splicing factor SRSF1 is essential for CD8 T cell function and host antigen-specific viral immunity
Source: Front Immunol. 2022 Sep 16;13:906355. doi: 10.3389/fimmu.2022.906355 (PMC9523749; doi:10.3389/fimmu.2022.906355)

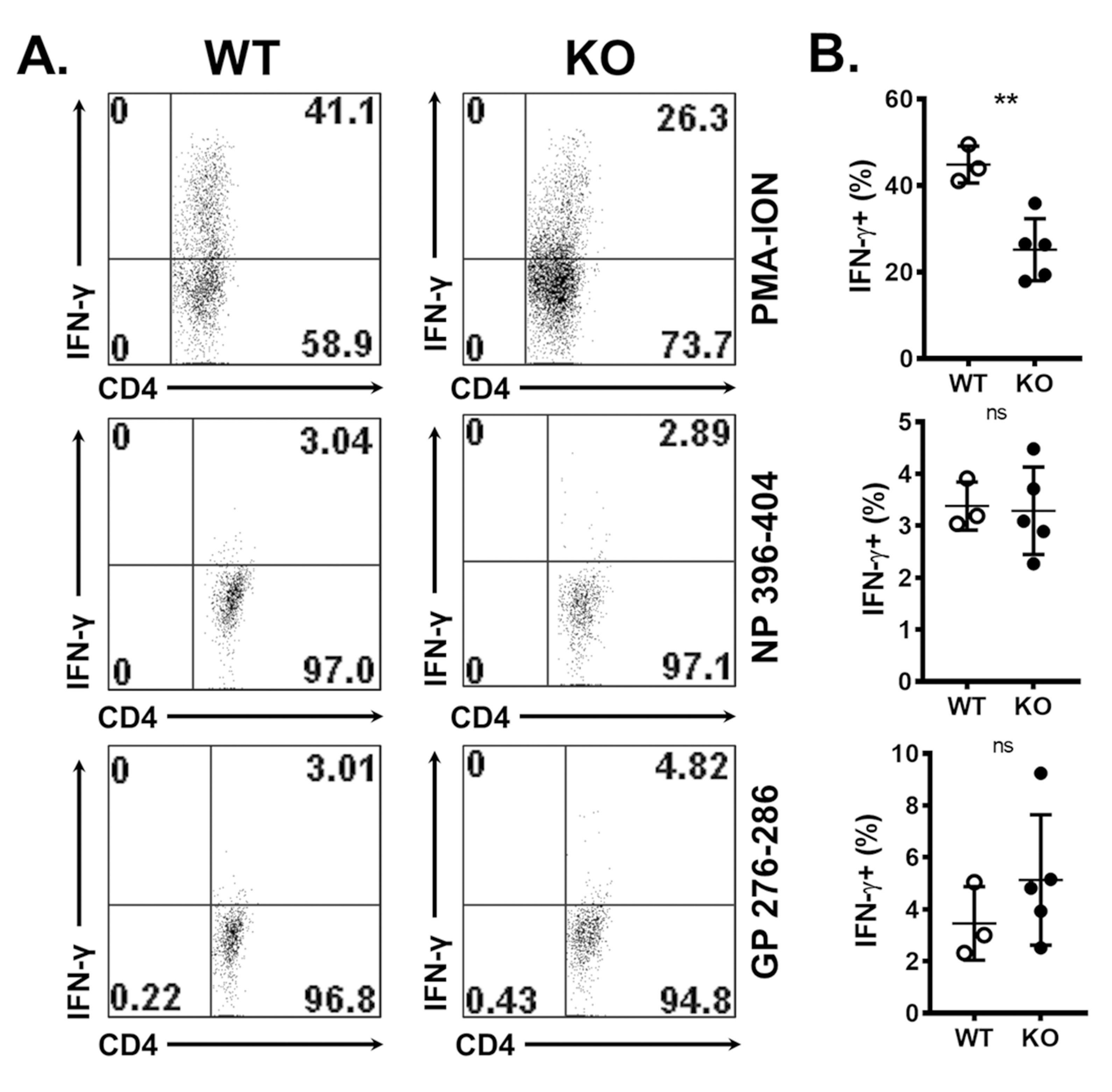

Supplement: Supplementary Figure 1 — SRSF1-KO CD4 T cells respond normally to LCMV peptides. (A) Plots show IFN-γ+ CD4 T cells from spleen cells stimulated with LCMV peptides NP 396-404 or GP 276-286 gated on Thy1.2+ T cells. [file Image_1.jpeg]

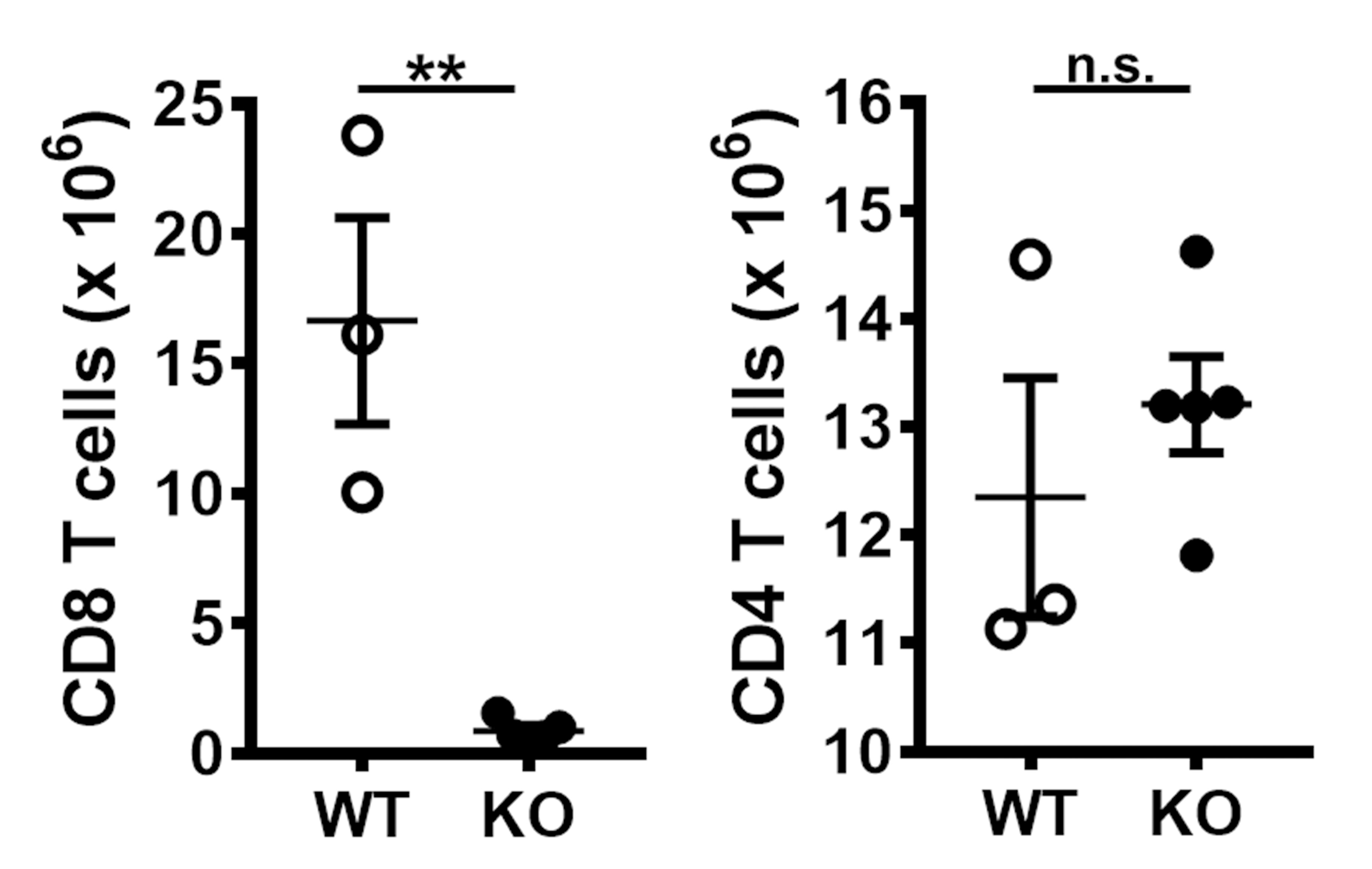

Supplement: Supplementary Figure 2 — CD8 and CD4 T cells numbers after LCMV infection (day 8). Graphs show the total numbers of CD8 and CD4 T cells in the spleen WT (n=3) and KO (n=5) mice. CD8 total number are depleted in KO mice (0.8 ± 0.2) compared to WT mice (16.6 ± 4.0) Graphs show mean ± SEM. p-values **: <0.01. [file Image_2.jpeg]

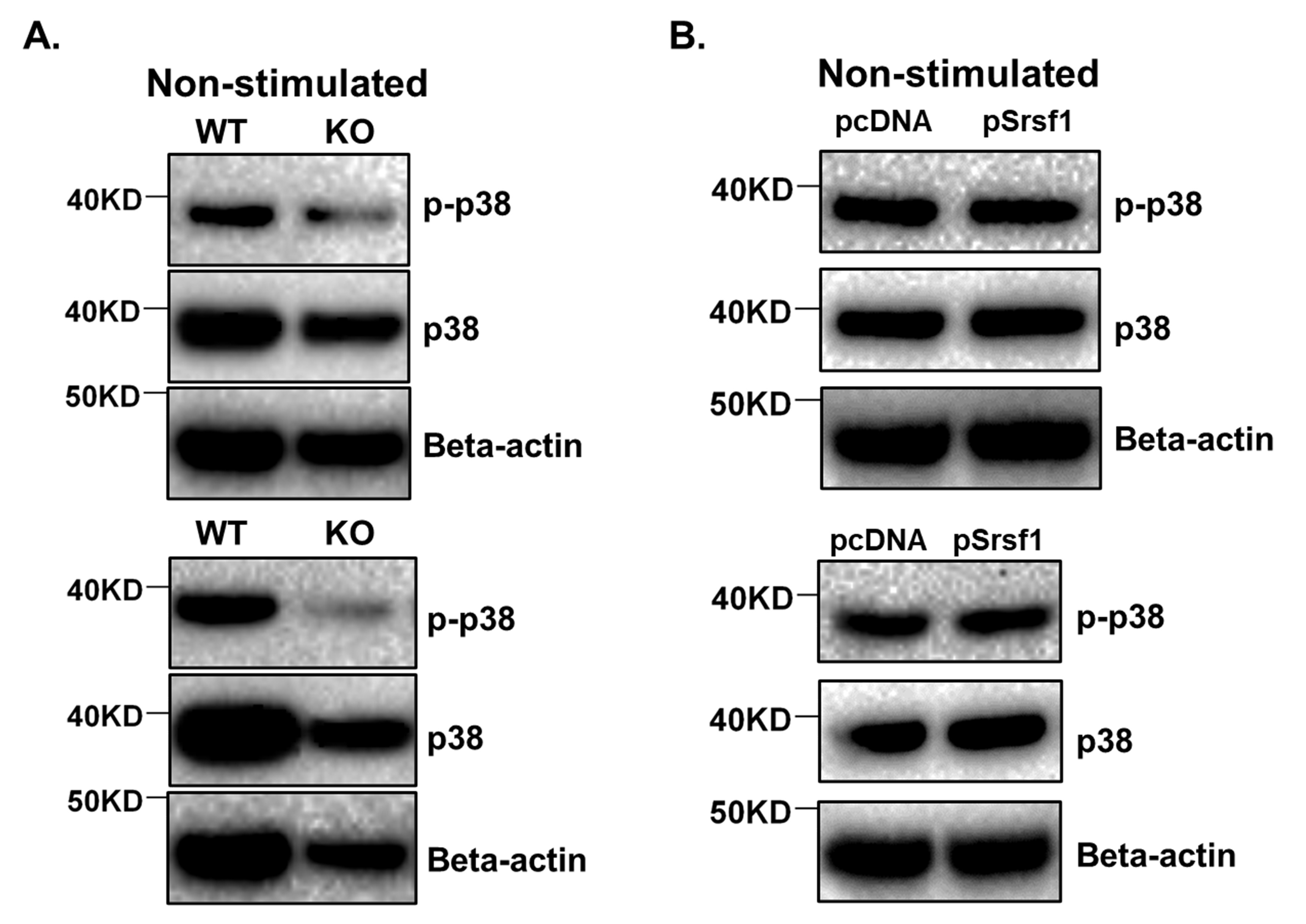

Supplement: Supplementary Figure 3 — Unstimulated control WB of Figure 6. (A) Total T cells were isolated from spleens of WT and Srsf1-cKO mice and, without stimulation, total protein was immunoblotted for phospho-p38 (p-p38), p38, and β-actin. Image showing two representative blot from 5 independent experiments. (B) Peripheral blood T cells were isolated from healthy donors and transfected with empty vector (pCDNA) or Srsf1 overexpression plasmid (pSrsf1). Unstimulated transfected T cells total protein was immunoblotted for phospho-p38 (p-p38), p38, and β-actin. Image showing two representative blot from 5 independent experiments. [file Image_3.jpeg]

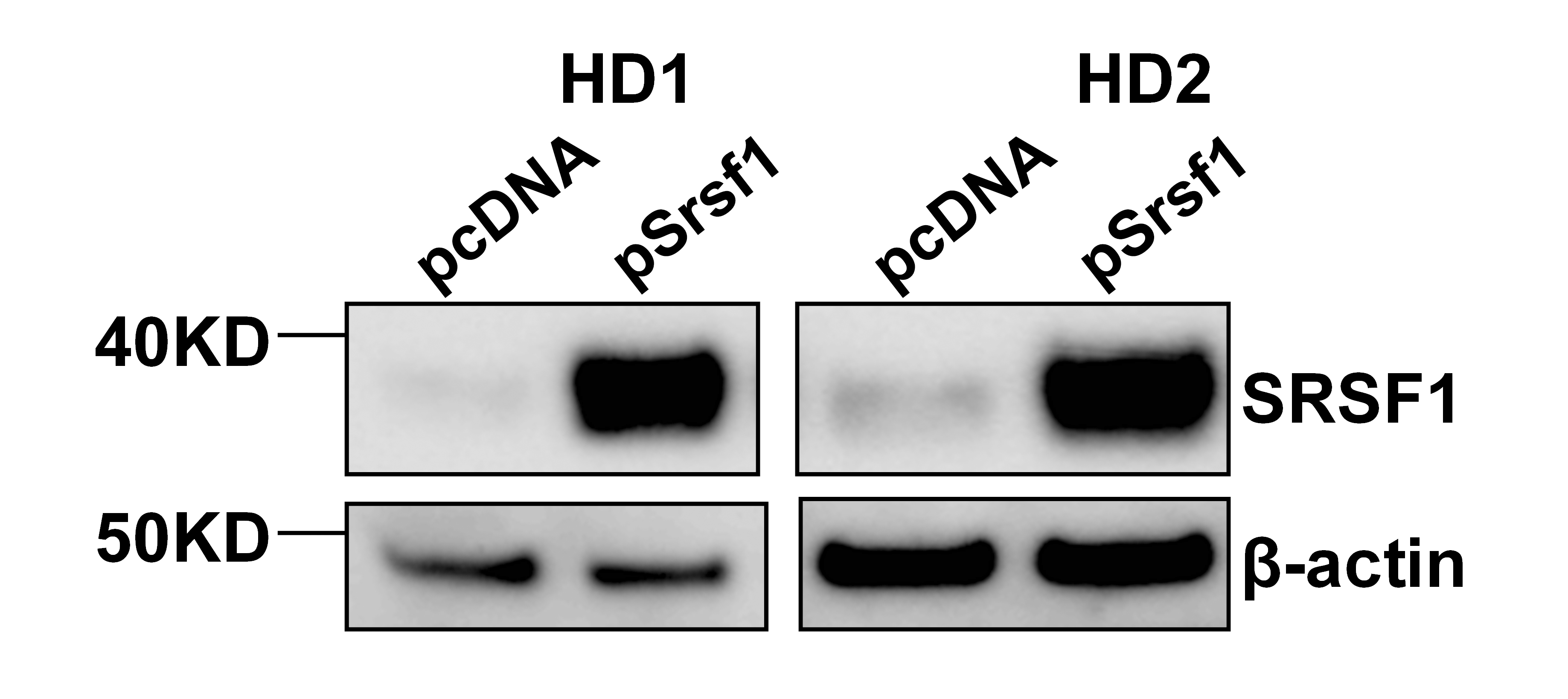

Supplement: Supplementary Figure 4 — SRSF1 overexpression in human T cells. Peripheral blood T cells were isolated from healthy donors (HD) and transfected with empty vector (pcDNA) or Srsf1 overexpression plasmid (pSrsf1). Total protein was immunoblotted for SRSF1 and β-actin. [file Image_4.jpeg]

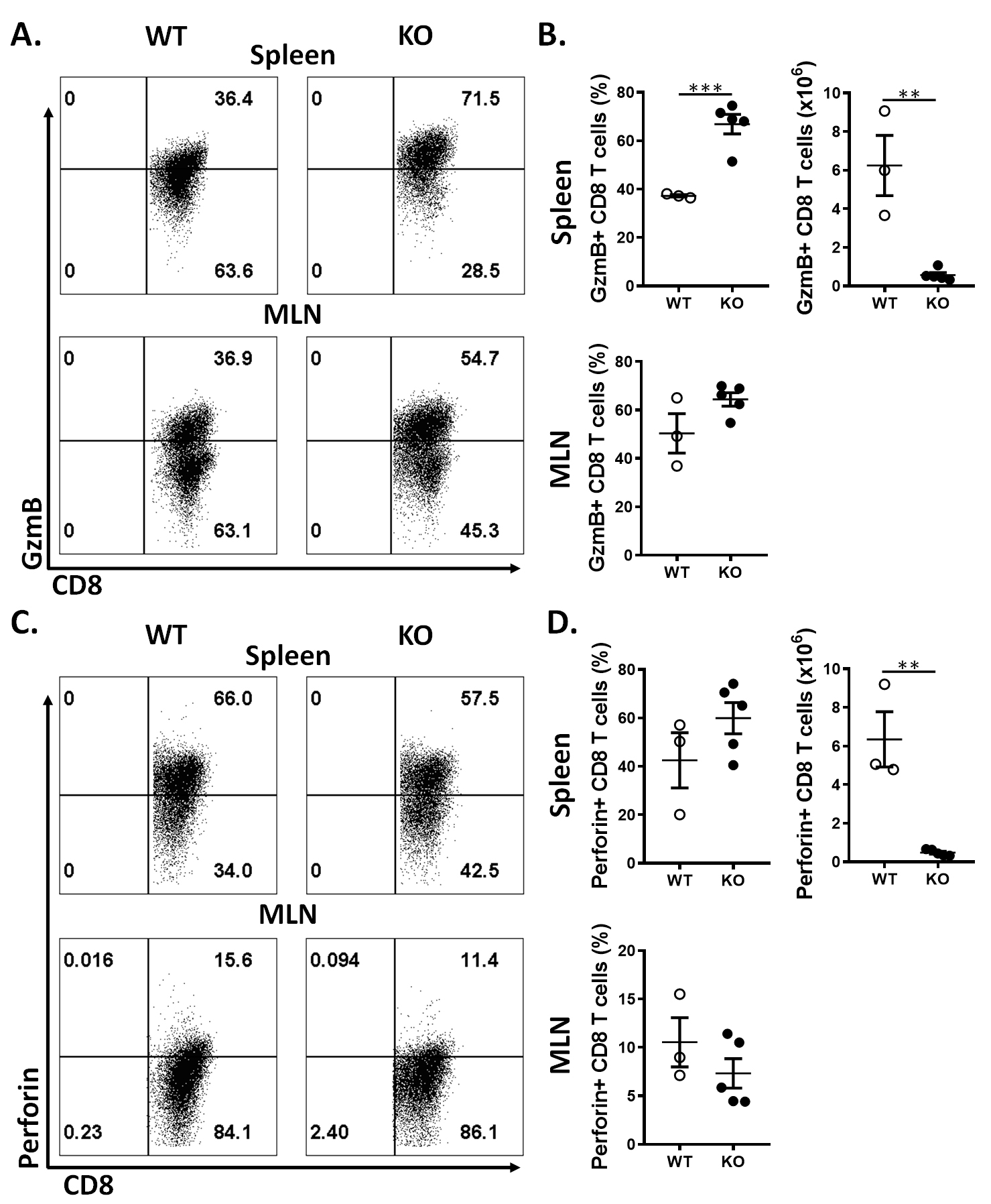

Supplement: Supplementary Figure 5 — Granzyme B+ and Perforin+ CD8 T cells are altered in SRSF1-KO mice following acute LCMV infection. (A) Flow cytometry dot plots show intracellular staining of granzyme B (GzmB) in CD8 T cells from the spleen and MLN of WT and SRSF1-KO (KO) mice. (B) Graphs show average frequencies of GzmB+ CD8 T cells in the spleen and MLN, as well as absolute numbers of GzmB+ CD8 T in spleens of WT and KO mice. (C) Flow cytometry dot plots show intracellular perforin staining in CD8 T cells from spleen and MLN of WT and KO mice. (B) Graphs show average frequencies of perforin+ CD8 T cells in the spleen and MLN, as well as absolute numbers of GzmB+ CD8 T cells in spleen, of WT and KO mice. ** = p<0.01; *** = p<0.001. [file Image_5.jpeg]
